# Meaning in life among nursing students: a latent profile analysis

**DOI:** 10.1186/s12912-023-01425-3

**Published:** 2023-08-11

**Authors:** Zhixin Zhao, Yongxia Mei, Xiaoxuan Wang, Hu Jiang, Wenna Wang, Beilei Lin, Zhenxiang Zhang

**Affiliations:** 1 School of Nursing and Health, Zhengzhou University, Henan, 450000 China; 2 School of Nursing, Inner Mongolia Medical University, Hohhot, 010000 Inner Mongolia China

**Keywords:** Latent profile analysis, Meaning in life, Nursing students, China, Nursing education

## Abstract

**Background:**

Meaning in life, defined by an individual’s understanding and appreciation of life, is a vital aspect of a positive psychological state, that has a significant influence on physical and mental health. Therefore, improving the sense of meaning in life among nursing students has emerged as a crucial concern in nursing education. This study aimed to clarify the profiles and influencing factors of meaning in life among nursing students.

**Methods:**

A descriptive cross-sectional online survey was conducted among nursing students in China from November 16, 2022, to January 17, 2023. The demographic information questionnaire and the meaning in life questionnaire (MLQ) were used to collect data. Latent profile analysis (LPA) was used to identify groups exhibiting distinct levels of meaning in life. Additionally, univariate analysis and multinominal logistic regression analysis were used to investigate the factors influencing each group. The reporting of this study adhered to the Strengthening the Reporting of Observational Studies in Epidemiology (STROBE) checklist.

**Results:**

A total of 10,583 valid responses were received, and the analysis revealed four distinct profiles. The profiles identified were the medium meaning group (C1, 41.4%), medium fluctuation meaning-no motivation group (C2, 8.7%), lower meaning group (C3, 9.7%), and higher meaning group (C4, 40.2%). The univariate analysis revealed that age, gender, ethnicity, marital status, educational level, grade, university classification, student leadership experience, and political affiliation were factors that influenced the four latent profiles (*P* < 0.05). The multinomial logistic regression analysis showed that age, gender, ethnicity, education level, and student leadership experience were significant predictors of the various profiles (*P* < 0.05).

**Conclusion:**

There is heterogeneous in meaning in life among nursing students in China. Nursing educators need to provide tailored guidance based on the latent classification characteristics of meaning in life among nursing students, aiming at improving their meaning in life and promoting the development of the nursing workforce.

**Supplementary Information:**

The online version contains supplementary material available at 10.1186/s12912-023-01425-3.

## Background

Nurses are the largest and the most professional group in the healthcare system [1], they support the stability of nursing workforce. Nurses are expected to have a healthy attitude, positive beliefs, and nursing competencies to provide excellent patient care [2]. However, they are experiencing a significant amount of stress at work, due to an increasing burden in addressing the complex needs of patients. Thus, the increasing number of nurses leaving their jobs each year, has become a global challenge [3]. The global demand for nurses is predicted to reach 5.9 million, and this issue can be addressed if the number of nursing graduates increases by 8% annually until 2030 [4]. For nurses, discovering the meaning of life can be a crucial strategy for addressing the issues and ensuring job retention [5]. The sense of meaning in life encompasses a positive mindset that significantly affects the physical health, mental health, and social functioning of an individual. As nursing students are the next generation of nursing professionals, it becomes the responsibility of nursing educators to promote a sense of meaning in life among them, while also ensuring their enjoyment of the nursing profession.

Frankl proposed the concept of a sense of meaning in life in 1963 [6], while Steger et al. [7] defined it in 2006 as people understanding and appreciating life, discovering the importance of their own lives, and recognizing the purpose and mission of life. Life’s sense of meaning has two dimensions: the presence of meaning in life and the search for meaning in life. The presence of meaning reflects the outcome or condition of an individual’s discovery of meaning, whereas the search for meaning reflects the process of an individual’s pursuit of meaning in life [8]. Meaning in life, as a positive psychological state, significantly influences the physical and mental health of individuals. Individuals who possess a stronger sense of meaning in life exhibit greater psychological resilience when faced with failures or tragedies [9, 10], and they adapt better to social life. Conversely, individuals lacking a sense of meaning in life, are more prone to emotions of emptiness and boredom. In severe cases, this can lead to a pervasive sense of apathy toward, everything in their surroundings, even to the point of disregarding life itself [11]. A study by Brassai et al. [12] found that a sense of meaning in life is a protective factor in maintaining and improving psychological well-being. It is also effective in preventing health risk behaviors to some extent. Moreover, when it comes to coping with stress, the meaningfulness of life can drive individuals to proactively face adversity by fostering positive emotions [13]. Furthermore, a strong sense of meaning in life not only improves interpersonal relationships, but also helps people improve their performance in academic and professional settings [14]. It helps them to identify with the activities they are indulged in, and take more initiative [15]. As nursing students, who will be part of the future professional group responsible for saving lives, they must cultivate a positive sense of meaning in life. By recognizing the goals, tasks, and missions of their own lives, nursing students can better appreciate the value and meaning of patients’ lives in their work.

As the future pool of nursing professionals, nursing students must possess a distinct sense of meaning in life. This sense of meaning in life enables them to affirm the value of life, appreciate the meaning of life, and discover the goal and direction of their lives, allowing them to be more actively involved in nursing work. However, several researchers have categorized nursing students into groups of high, medium, and low meaning in life based on the total scores or score rates. These classifications resulted in simplified regarding the influencing factors of meaning in life among nursing students. This approach cannot adequately reflect the differences in the meaning of life among nursing students at an individual level. Latent profile analysis (LPA) is an individual-centered method that clusters data using continuous explicit variables, allowing for the exploration of population heterogeneity [16]. Furthermore, the person-centered method focuses on identifying latent subgroups of persons based on multiple observed characteristics (i.e., indicators), giving this approach a higher level of specificity than the variable-centered approach [17]. The variable-centered approach, overlooks individual experiences, presenting only a generalized or average image [18]. To address this limitation, the present study aims to employ a person-centred approach to investigate the potential categories of meaning in life among nursing students in universities and the differences in their characteristics using latent profile analysis. This analysis will provide valuable insights for tailored interventions aimed at enhancing meaning in life among nursing students.

## Methods

### Design

A descriptive cross-sectional design was adopted. This study’s reporting followed the reporting of observational studies (STROBE) checklist (see Supplementary file 1).

### Participants

A total of 10,583 nursing students in China were recruited by convenient sampling from November 16, 2022, to January 17, 2023. The sample size for this study was determined to be at least 500, as LPA requires a sample size greater than 500 [19]. The inclusion criteria for participants were as follow: (1) being at least 16 years old and enrolled as a full-time students in China; (2) having internet accessibility; (3) having adequate cognitive and behavioral abilities, and (4) being willing to provide informed consent and participate in this study. Nursing students who were diagnosed with a major psychiatric issue or mental illness and those on sabbatical from school were excluded from the study.

### Data collection

The questionnaire were administered through the ‘‘Questionnaire Star” network platform (www.wjx.cn), a popular online data-gathering tool in China used for conducting web surveys. WeChat, a well-known social media platform, was used to distribute both the QR code and the link to the online questionnaire. The questionnaire started with a section that explained the purpose, significance, and instructions for completing the survey before the formal questions. Each IP address can only be entered once to avoid duplication.

### Instruments

#### Participant characteristics

The demographic information questionnaire for this study was specifically developed by the researchers and included such as age, gender, ethnicity, marital status, family residence, education level, grade, university classification, student leadership experience and political affiliation.

### The meaning in life questionnaire (MLQ)

The meaning in life questionnaire (MLQ) developed by Steger et al. [7] is a simple scale that assesses two dimensions: the presence of meaning and the search for meaning. These dimensions measures individuals’ tendency to pursue the value of life and the perceived purpose of and value of life, respectively. In this study, the Chinese version of the MLQ, translated by Liu et al. [20] in 2010 was used. This version consists of nine items. The Likert-seven scoring system was used, with the scoring scale ranging from “completely non-compliant” (score of 1) and “completely compliant” (score of 7), and the total score ranges from 9 to 63. A higher score indicates higher levels of meaning in life. In this study, Cronbach’s alpha of the questionnaire was 0.80, and that of presence and search for meaning were 0.81 and 0.72, respectively.

### Ethical considerations

This study was approved by the Ethics Committee of Zhengzhou University (ZZUIRB2021-918), and permission for the data collection was obtained from all participants. Upon accessing the survey link, nursing students were presented with an informed consent form, outlining the purpose, significance, inclusion, and exclusion criteria of this study. Participants were informed that their participation was voluntary and confidential, and that they could withdraw from the study at any time without providing a reason or facing any consequences.

### Data analysis

Mplus 7.4 was applied to analyze the latent profile based on nine items of meaning in life. The evaluation indexes of the latent profile model included the Akaike information criterion (AIC), Bayesian information criterion (BIC) and adjusted Bayesian information criterion (aBIC). Lower values of AIC, BIC, and aBIC indicate a better fit of the model [21]. Additionally, information Entropy > 0.8 (on a scale of 0 to 1) indicates a classification accuracy exceeding 90% [22, 23]. The Bootstrap likelihood ratio test (BLRT) and the Lo-Mendell-Rubin likelihood ratio test (LMRT) were used to compare the fit of different class models. If the *P*-values corresponding to LMRT and BLRT reached a significant level, the K-class model was deemed superior to the K-1 class model [19, 24]. Furthermore, the average attribution probability matrix was examined, and if the values on the diagonal were all higher than 0.7, it indicated an acceptable classification of the model [25]. SPSS 26.0 was used for descriptive analysis. Categorical data were described using frequencies and percentages (%), while quantitative data were presented as mean ± standard deviation ($\overline x$± SD). Univariate analysis and multinominal logistic regression analysis were performed to evaluate the influence of various factors on the categories of meaning in life among the participants. A *P*-value < 0.05 indicated a statistically significant difference.

## Results

### Participants’ characteristics

A total of 10,756 individuals responded to online questionnaires. After excluding 173 individual with incomplete information, a total of 10,583 participants were included in the study, resulting in a 98.4% response rate. Among the included participants, 85.5% were females. The mean age of the students was 19.51 (SD 1.83, range 17–40) years. The majority of participants resided in a rural areas (79.1%). More detailed information can be found in Table 1.

**Table 1:** General information of the participants (*N* = 10,583)

Characteristics		n (%)
Gender	Male	1538(14.5)
	Female	9045(85.5)
Ethnicity	Han	9961(94.1)
	Ethnic Minority	622(5.9)
Marital Status	Unmarried	10,450(98.7)
	Married	133(1.3)
Family residence	Rural	8370(79.1)
	Urban	2213(20.9)
Education level	Junior college	8459(79.9)
	Bachelor’s	1886(17.8)
	Master’s	229(2.2)
	Doctor’s	9(0.1)
Grade	Freshman	4924(46.5)
	Sophomore	3651(34.5)
	Junior	1062(10.0)
	Senior	203(1.9)
	Fifth grade and above	740(7.0)
University classification	Associate college	8238(77.8)
	General Undergraduate	2107(19.9)
	Double -class institution	238(2.2)
Political affiliation	Communist Party member	323(3.1)
	Communist Youth League Member	5679(53.7)
	General public	4530(42.8)
	others	51(0.5)
Student leadership experience	Yes	3393(32.1)
	No	7190(67.9)

### LPA results of meaning in life among participants

In this study, nine items of the meaning in life questionnaire were used as explicit indicators, and a 1–5 latent profile model was selected to conduct the exploratory latent profile analysis of the meaning in life of the participants (Table 2). The results show that: As the number of categories gradually increased, the values of AIC, BIC, and aBIC for models 1–5 continuously decreased. Additionally, the values of LMRT and BLRT were all statistically significant. In the four-category model, the entropy value was 0.939, which is higher compared to the entropy value of 0.891 in the five-category model. A higher entropy value, closer to 1, indicates a higher classification accuracy. In addition, the value of the diagonal on the average probability matrix of the latent categories was significantly higher than 0.70, demonstrating that the results of the four-category latent classification model are reliable (Table 3).

**Table 2:** Fitting index of latent profile analysis about the participants’ meaning in life

	AIC	BIC	aBIC	Entropy	LMRT	BLRT	Class Probability
1	370075.646	370206.452	370149.250	-	-	-	1
2	338203.727	338407.203	338318.223	0.893	< 0.01	< 0.01	0.35/0.65
3	318765.510	319041.657	318920.898	0.925	< 0.01	< 0.01	0.09/0.46/0.45
4	312122.977	312471.794	312319.256	0.939	< 0.01	< 0.01	0.41/0.09/0.10/0.40
5	307942.404	308363.890	308179.574	0.891	< 0.01	< 0.01	0.08/0.09/0.23/0.27/0.33

AIC, Akaike information criterion; BIC, Bayesian information criterion; aBIC, sample size-adjusted Bayesian information criterion; BLRT, bootstrap likelihood ratio test; LMR, Lo–Mendell–Rubin adjusted likelihood ratio test

**Table 3:** Average attribution probability matrix for each potential profile

Potential Profile	1	2	3	4
1	0.963	0.009	0.004	0.024
2	0.041	0.937	0.001	0.021
3	0.019	0.002	0.979	0.000
4	0.024	0.004	0.000	0.972

### Latent category and characteristics of meaning in life among the participants

**Fig. 1:**
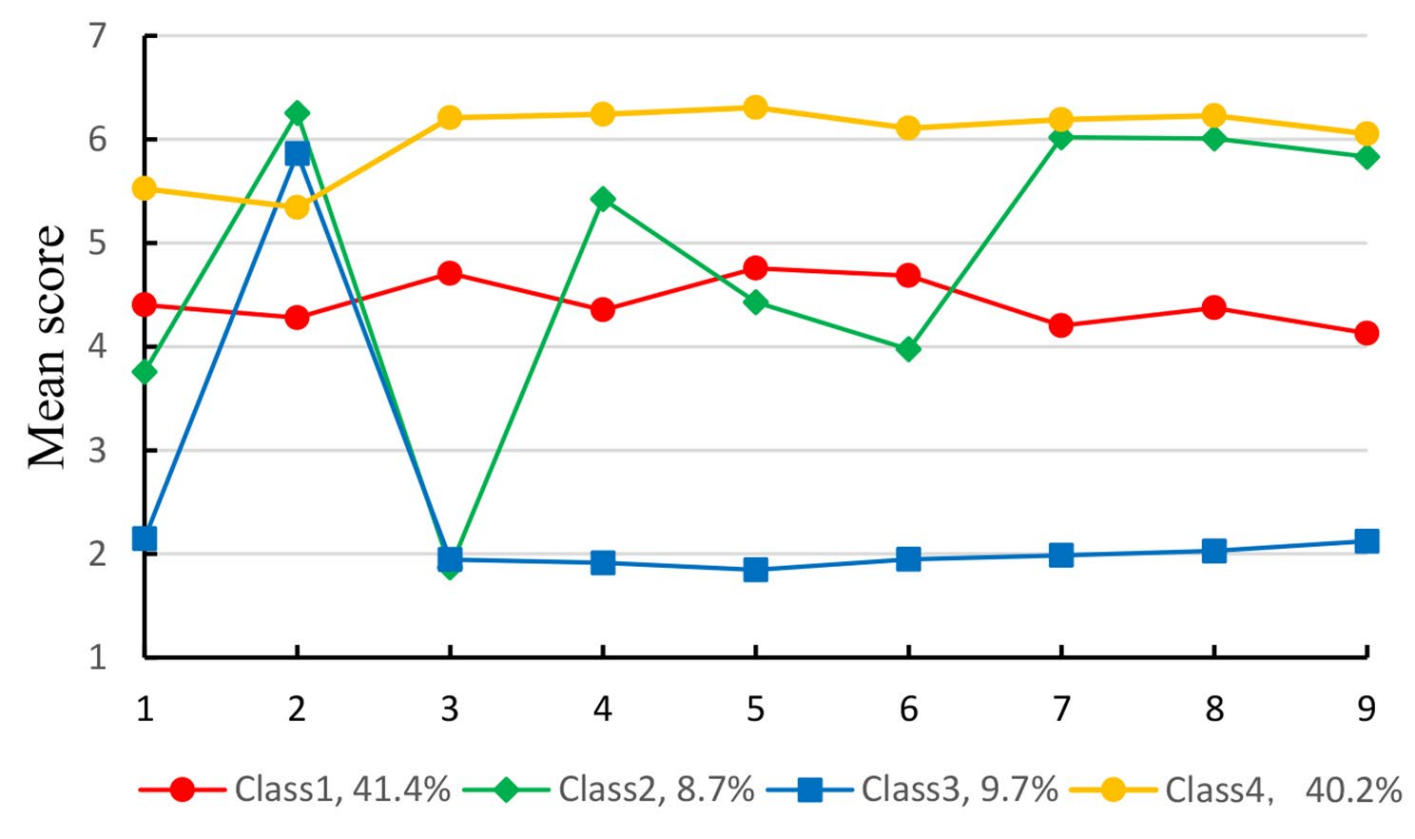
The participants’ scores in each item of the MLQ

Based on the latent classification results, the researchers plotted the scores of the four latent categories on each topic of the MLQ. See Fig. 1.

The total score for C1 was 39.31 ± 4.51, and the average score for all items was approximately 4 points, indicating a medium level of meaning in life. Therefore, this category was named the “medium meaning group”. There were 4,382 participants in this group (41.4%). The total score for C2 was 39.09 ± 5.64, also indicating a moderate level of meaning in life. However, there was a wide range of fluctuations in each item with these category. In C2, the scores were high for Item 2 (I have no clear purpose in my life), Item 4 (I understand the meaning of my life), Item 7 (I have a clear direction in my life), Item 8 (I know what makes my life meaningful), and Item 9 (I have found a life purpose that satisfies me). All scores were above 5 and Item 2 is a reverse scoring question, indicating that this category of nursing students does not lack a purpose in life. The score for Item 3 (I am searching for the meaning of my life) was low, below 2. However, Item 6 (I am always trying to find the purpose of my life) had scores above 4. This group of nursing students can perceive the meaning in life but lacks the motivation to actively pursue it. As a result, it was named the “medium fluctuation meaning-no motivation group”, with a total of 921 individuals, accounting for 8.7% of the sample size. The total score for C3 was 18.09 ± 6.36, and the average score for each item was above 2. Notably, Item 2 (My life does not have a clear purpose) had scores above 6, indicating that this group of nursing students have a clear purpose in life but struggle to perceive meaning in life. It was named the “lower meaning group”. There were 1,030 nursing students in this group, accounting for 9.7% of the participants. The total score for C4 was 51.57 ± 4.72, with an average score of approximately 6 for each item. This indicates that nursing students in this group have a clear purpose in life and accurately perceive the meaning of life. Consequently, this group was named the “higher meaning group”, and comprised a total of 4,250 people, accounting for 40.2% of the participants.

### Univariate analysis and multivariate logistic regression analysis of the factors influencing the latent categories of meaning in life among participants

In the univariate analysis, age, gender, ethnicity, marital status, educational level, grade, university classification, student leadership experience, and political affiliation were factors that influenced the four latent profiles (*P* < 0.05). The results were presented in Table 4.

In the disordered multi-classification logistic regression, using C4 as the reference group, the results showed that the statistically significant influencing factors were age, gender, ethnicity, education level, and student leadership experience. See Table 5.

The comparison between the C1 and C4 revealed the following findings: (1) the likelihood of nursing students belonging to C1 decreased by 6% with decreasing age (*P* < 0.05); (2) Han nursing students had a 1.44 times higher likelihood of belonging to C1 compared to nursing students from ethnic minorities (*P* < 0.05); and (3) the likelihood of belonging to C1 decreased by 15% among nursing students with student leadership experience compared to those without student leadership experience (*P* < 0.05).

The comparison between the C2 and C4 yielded the following results: (1) male nursing students had a 1.27 times higher likelihood of belonging to C2 compared to female nursing students (*P* < 0.05); (2) the likelihood of belonging to C2 was reduced by 94% (*P* < 0.05), 95% (*P* < 0.05), and 95% (*P* < 0.05) for nursing students with an associate, undergraduate, and master’s degree, respectively, compared to those with a doctoral degree,.

The comparison between the C3 and C4 revealed that male nursing students had a 1.44 times higher likelihood of belonging to C3 compared to female nursing students (*P* < 0.05).

**Table 4:** Demographic information four profile latent profiles among the participants (n,%)

Variables	Class 1 (n = 4382)	Class 2 (n = 921)	Class 3 (n = 1030)	Class 4 (n = 4250)	χ^2^/*F*	*P*
Age	19.42 ± 1.66	19.44 ± 1.77	19.56 ± 1.80	19.62 ± 1.20	9.985	＜0.001
Gender					38.931	＜0.001
Male	525 (16.4)	153(16.6)	211(20.5)	587(13.8)		
Female	3795(86.6)	768(83.4)	819(79.5)	3663(86.2)		
Ethnicity					22.213	＜0.001
Han	4180(95.4)	855(92.8)	958(93.0)	3968(93.4)		
Ethnic Minority	202(4.6)	66(7.2)	72(7.0)	282(6.6)		
Marital status					13.979	0.003
Unmarried	4348(99.2)	906(98.4)	1013(98.3)	4183(98.4)		
Married	34(0.8)	15(1.6)	17(1.7)	67(1.6)		
Family residence					7.323	0.062
Rural	3489(79.6)	710(77.1)	838(81.4)	3333(78.4)		
Urban	893(20.4)	211(22.9)	192(18.6)	917(21.6)		
Education level					76.830	＜0.001
Junior college	3494(79.7)	780(84.7)	883(85.7)	3302(77.7)		
Bachelor’s	807(18.4)	120(13.0)	133(12.9)	826(19.4)		
Master’s	79(1.8)	17(1.8)	12(1.2)	121(2.8)		
Doctor’s	2(0.1)	4(0.4)	2(0.2)	1(0.1)		
Grade					38.032	＜0.001
Freshman	2036(46.5)	453(49.2)	412(40.0)	2023(47.6)		
Sophomore	1528(34.9)	298(32.4)	400(38.8)	1425(33.5)		
Junior	450(10.3)	72(7.8)	105(10.2)	435(10.2)		
Senior	85(1.9)	20(2.2)	18(1.7)	83(2.0)		
Fifth grade and above	283(6.5)	78(8.5)	95(9.2)	284(6.7)		
University classification					41.894	＜0.001
Associate college	3413(77.9)	767(83.3)	767(83.3)	3213(75.6)		
General Undergraduate	865(19.7)	134(14.5)	172(16.7)	936(22.0)		
Double-class institution	104(2.4)	20(2.2)	13(1.3)	101(2.4)		
Political affiliation					35.800	＜0.001
Communist Party member	110(2.5)	28(3.0)	31(3.0)	154(3.6)		
Communist Youth League Member	2294(52.4)	491(53.3)	518(50.3)	2376(55.9)		
General public	1953(44.6)	400(43.4)	472(45.8)	1705(40.1)		
Others	25(0.6)	2(0.2)	9(0.9)	15(0.4)		
Student leadership experience					96.908	＜0.001
Yes	1173(26.8)	341(37.0)	366(35.5)	1513(35.6)		
No	3209(73.2)	580(63.0)	664(64.5)	2737(64.4)		

**Table 5:** Multivariate Logistic regressions for predicting in four profile latent classes among the participants

Variables	Class 1 VS Class 4				Class 2 VS Class 4				Class 3 VS Class 4		
	*β*	*OR*	95%*CI*		*β*	*OR*	95%*CI*		*β*	*OR*	95%*CI*
Age	-0.058	0.944**	0.906–0.983		-0.039	0.961	0.907–1.020		-0.029	0.971	0.910–1.036
Gender											
Male	0.059	1.061	0.918–1.226		0.242	1.273*	1.043–1.555		0.362	1.437**	1.151–1.794
Female (refer)											
Ethnicity											
Han	0.362	1.437**	1.159–1.782		-0.055	0.946	0.711–1.260		0.044	1.045	0.750–1.456
Ethnic Minority (refer)											
Marital status											
Unmarried	0.035	1.035	0.602–1.780		-0.412	0.662	0.324–1.355		-0.748	0.473	0.211–1.062
Married (refer)											
Family residence											
Rural	-0.040	0.961	0.853–1.083		-0.119	0.888	0.746–1.058		0.107	1.113	0.901–1.374
Urban (refer)											
Education Level											
Junior college	-1.091	0.336	0.027–4.203		-2.892	0.055*	0.006–0.536		-1.734	0.177	0.009–3.576
Bachelor’s	-0.995	0.370	0.030–4.603		-3.038	0.048**	0.005–0.461		-2.430	0.088	0.004–1.764
Master’s	-1.108	0.393	0.026–4.189		-3.063	0.047*	0.005–0.473		-2.606	0.074	0.003–1.576
Doctor’s (refer)											
Grade											
Freshman	-0.047	0.954	0.778–1.170		-0.171	0.842	0.637–1.114		-0.274	0.761	0.555–1.043
Sophomore	0.134	1.144	0.929–1.408		-0.179	0.837	0.628–1.115		-0.008	0.992	0.721–1.364
Junior	0.141	1.151	0.892–1.487		-0.252	0.778	0.533–1.133		-0.058	0.943	0.629–1.416
Senior	0.090	1.095	0.730–1.641		0.174	1.190	0.666–2.123		-0.026	0.974	0.493–1.925
Fifth grade and above (refer)											
University classification											
Associate college	-0.172	0.842	0.538–1.318		0.067	1.070	0.532–2.152		-0.292	0.747	0.329–1.694
General Undergraduate	-0.209	0.812	0.575–1.145		-0.259	0.772	0.445–1.338		0.295	1.343	0.650–2.778
Double-class institutions (refer)											
Political affiliation											
Communist Party member	-0.432	0.649	0.280–1.506		0.475	1.608	0.338–7.660		-0.056	0.946	0.286–3.129
Communist Youth League Member	-0.379	0.685	0.311–1.507		0.444	1.559	0.349–6.969		-0.129	0.879	0.297–2.599
General public	-0.295	0.744	0.338–1.640		0.434	1.544	0.345–6.905		-0.032	0.969	0.327–2.868
Others (refer)											
Student leadership experience											
Yes	-0.166	0.847**	0.761–0.943		0.132	1.141	0.977–1.332		0.174	1.190	0.996–1.421
No (refer)											

*p < 0.05, **p < 0.01; *OR*, Odds ratio; *95% CI*, 95% Confidence Interval

## Discussion

### Nursing students exhibit group heterogeneity in their levels of meaning in life, the majority falling at the medium level

The participants in this study demonstrated a medium level of meaning in life, with an average score of 42.15 ± 10.96. Previous study have found that a higher level of meaning in life enhances resilience to stress and generally improves physical health [26]. Meaning in life enables nursing students to effectively cope with stress and recover from adversity [27]. However, the findings of this study revealed lower scores compared to Huang Jiali [28] and Gao Ran et al. [29] regarding nurses’ sense of meaning in life. This difference could be attributed to the fact that nursing students, who have not yet entered the workforce, may place less emphasis on the concept of meaning in life. The findings of this study revealed higher scores compared to Yao Mengping et al.‘s [30] survey on college students’ sense of meaning in life. This difference may be attributed to the fact that nursing students are exposed to life-related education that prompts them to think more about life.

The LPA revealed four latent categories of meaning in life, reflecting individual heterogeneity among nursing students. These categories are characterized by varying scores and notable features in specific items. One such category is the “medium fluctuating meaning-no motivation group (C2)” which exhibits moderate level scores. However, unlike the C1 group, this group has a greater difference in scores for certain items. Students belonging to this category have a clear purpose in life, but lack the motivation to actively pursue meaning in life. Although this group accounts for only 8.7% of the sample size, it should be given sufficient attention. Additionally, the “lower meaning group (C3)” exhibits a lack of sense of meaning in life,reflected in lower scores on both dimensions of meaning in life (presence of meaning in life and search for meaning in life). Approximately 9.7% of individuals in this group cannot perceive meaning in life, highlighting the need for an enhanced education on the value of life. The “higher meaning group (C4)” exhibits a high level of meaning in life. Individuals belonging to this group can accurately perceive the meaning in life and are highly motivated to search for meaning in life. A clear self-concept is of utmost importance for nursing students. Research indicates a positive correlation between self-concept clarity and meaning in life [31]. Nursing students with a high level of self-concept clarity tend to experience a deep self-understanding and a greater sense of meaning in life [32]. Additionally, individuals with a high meaning in life tend to experience a greater sense of happiness in their lives [33], which can further contribute to their professional development and achievements [34]. By analyzing the heterogeneity among nursing students, educators can gain a precise understanding of their ideological dynamics. This insight enables them to implement timely and tailored meaning-oriented programs and career planning initiatives, fostering a re-evaluation of nursing, and the development of correct life concepts and attitudes among students.

### Factors influencing latent categories of meaning in life among nursing students

In the current study, age emerged as an important factor that affects the sense of meaning in life. Some studies have shown that meaning in life becomes more positive with age [35]. Steger et al. [36] found that meaning in life varies among different age groups. As individuals grow older, the presence of meaning and search for meaning change. Specifically, the motivation to explore meaning in life tends to decrease, while the ability to perceive meaning in life increase.

Gender emerged as a factor influencing the sense of meaning in life, but there is still no consensus on the specific impact of different genders on the sense of meaning in life. A study by Dong xiuzhi [37]on oncology nursing staff showed that male nurses experience a greater sense of meaning in life. This finding can be attributed to the presence of traditional gender stereotypes deeply ingrained in society. These stereotypes hold that men should be more aggressive and responsible. Additionally, these stereotypes encourage men to rediscover the purpose of their lives and have a clearer orientation toward their gender roles. Some studies suggest that females experience a greater sense of meaning in life. There are a couple of reasons of this. Firstly, females are more likely to connect and communicate with others and have a diverse social relationships, which can contributed to an increased feeling of purpose in life [38]. Secondly, considering the nursing field, which predominantly attracts a larger number of female students, it may pose challenges for males to identify themselves within this predominantly female group, potentially impacting their sense of meaning in life. However, it is important to acknowledge that the significant difference in male-to-female ratios in this study may have limited the scope of conclusions that can be drawn. Further research is needed to explore this topic more comprehensively in the future.

In this study, ethnicity emerged as a predictor of belonging to C2. Specifically, Han nursing students were more likely to exhibit a moderate sense of purpose in life compared to students from other ethnic backgrounds. This finding may be due to cultural considerations. According to Geertz [39] “culture is the web of meaning woven by the person,“ and the cultural heritage of ethnic groups reflects the diversity of their culture and meaning in life constructs.

Education influenced the sense of meaning in life among students in C2. There was no significant difference observed in the other categories in terms of education level, which is consistent with the findings of Hsiao et al. [40]. That could be attributed to the fact that, upon entering college, students in various degree programs have distinct academic focuses, greater autonomy in decision-making and diverse perspectives regarding the significance of their lives.

Student leadership experience influenced the sense of meaning in life. Being involved as student leaders allows individuals to build relationships with their peers and engage in positive interpersonal interactions, leading to feelings of safe, warmth, value, and meaning in life. This fosters the growth of positive personality traits and life values. According to a study by Du Li et al. [38] on the sense of meaning in life among practicing nursing students, undergraduate nursing students with strong interpersonal connections reported higher levels of meaning in life.

### Practical implications

Cultivating a sense of meaning in life is crucial for enhancing the psychological well-being of nursing students [41, 42]. Consequently, it is imperative to monitor meaning in life among this group. This study holds significant implications for the field of higher education in nursing by providing an idea and classification strategy. Nursing administrators and educators can utilize these findings to develop individualized approaches to life education for nursing students with unique potential profile characteristics.

The use of LPA in identifying distinct life meaning profiles among nursing students offers valuable insights for the development of personalized intervention programs. This approach allows for interventions that are better tailored to the specific needs of individual nursing students [43]. In recent years, several scholars have conducted studies focused on the death education curriculum, primarily targeting undergraduate nursing students or clinical nurses. These studies indicate that the establishment and improvement of the curriculum system need to be adapted to the physical and mental development characteristics of the study subjects [44]. This present study found four different profiles of nursing students’ meaning in life, with different influencing factors for each subgroup. The findings of this study help nursing educators to personalize and strengthen education on the value of life and establish a scientifically accurate view of life and death, considering the characteristics of different educational levels, grades, and genders when conducting curriculum education. Moreover, research has demonstrated that discovering meaning in life increases the nursing students’ likelihood of understanding human existence and strengthens commitment to assisting patients in finding meaning in their own lives [45, 46]. Through latent profile analysis, nursing students have a clear understanding of which subgroup they belong to, so they can make changes for the development of further nursing practice.

### Limitations

There were several limitations in this study. First, our research was based on a self-reported web-based survey and convenience sampling limiting the generalizability of the findings. Second, female students constituted the majority of the sample size, decreasing the generalizability of the findings. Third, this study was a cross-sectional study and no causal association could be established. Therefore, a longitudinal study should be performed to replicate these findings.

### Contributions to the nursing field

This study will be able to offer an idea and classification strategy to be used in the field of higher education in nursing, as it provides nursing administrators and educators with methods to develop an individualized approach to life education for nursing students with different potential category characteristics. This initiative helps nursing students to find meaning in their lives and careers. Discussing this issue with future health professionals can lead to better personal perspectives and options for further nursing practice.

## Conclusion

Meaning in life plays a significant role in shaping nursing students’ future career development and realization of their life value. As nursing is a deeply humanistic profession, it is crucial to enhance the life value education for nursing students. This study is the first effort to identify subtypes of meaning in life among nursing students using latent profile analysis to designate categories. The results provide new insights for nursing educators and policymakers to design targeted life education activities. Nursing students with different potential categories of meaning in life can take personalized training and guidance.

### Electronic supplementary material

Below is the link to the electronic supplementary material.

Supplementary Material 1

## Data Availability

The data supporting the findings of this study are available on request from the corresponding author. The data are not publicly available due to privacy or ethical restrictions.
